# Supplementary material for: On the possibility of oscillating in the Ebola virus dynamics and investigating the effect of the lifetime of T lymphocytes
Source: PLoS One. 2022 Mar 11;17(3):e0265065. doi: 10.1371/journal.pone.0265065 (PMC8916666; doi:10.1371/journal.pone.0265065)
Supplement: Attachment — Submitted filename: Response to Reviewers.docx [file pone.0265065.s002.docx]

Please include the following items when submitting your revised manuscript:

A rebuttal letter that responds to each point raised by the academic editor and reviewer(s). You should upload this letter as a separate file labeled 'Response to Reviewers'.

We have responded to the reviewers’ comments point-by-point. The reviewers' comments are in red. Our responses have been written as black text. We also highlighted the changes in the revised version of the manuscript in yellow.

Reviewer #1: The authors of “On the Possibility of Oscillating in the Ebola Virus Dynamics and Investigating the Effect of the Lifetime of T lymphocytes” describe a cellular automata (CA) model to represent the spread of Ebola virus in lymph nodes. While this is an important topic and the authors present interesting results, there are some questions and concerns about the model, as well as potentially some missed opportunities to take advantage of what CA models can offer.

1. One strength of CA models is to offer spatial insights, yet there are no spatial results given beyond figure 2. One important question arising from their results is: what does the spatial distribution of infected cells look like in simulations that oscillate?

Thanks for the reviewer’s helpful comment. We totally agree with the reviewer. So, we added **Figure 11** in the manuscript which demonstrates snapshots of one of the samples which took at every 20 time-steps. Besides, a paragraph was added for explaining this Figure in the “**Results and Discussion**” section (**page 11**).

“The snapshots of one of the samples which are taken at every 20 time steps are shown in Figure 11. The simulation started by inserting one infected cell in the centre of the lattice. In this case, we set *P_I_*=1 which means the contamination ability of the infected cells is at its maximum value. The ability of movements of the cells makes the chance to the infected cells to come close to the healthy cells and convert them to the infected cells. In contrast, the number of T cells will increase in the centre of the lattice because of the value of the probability of creation of a T cell is much more than the value of the probability of their death. These two mechanisms make the possibility of formation of moving circular waves with infected cells at their boundary and large numbers of T cells at the inner layers. By passing time, the size and the number of these waves decrease and increase respectively.”

2. Another missed opportunity lies in comparing results between the ODE and CA models (which the authors state that they will do (p4), and mention again on p16) but do not present any results for). It would be interesting to see if the oscillations arise because of the spatial nature of CA models, or if this is something that is preserved from the ODE model?

Thanks for the reviewer’s useful comment. We added some sentences in the “**Conclusion**” section (**page 14**) which mentioned that we compared our results with the results of model proposed by Wester et al.

“We also compared our results with the results of an ODE model proposed by Wester et al. (8). In general, they used combination of some parameters for generating their data and because of that, an exact and one by one comparisons of their results with our CA results are not possible. However, some similarity could be found. For example, in Figure 8 of their work, an oscillation dynamics behaviour could be also seen which is in concordance with an oscillation behaviour in the CA model.”

3. Another key feature of CA models is their stochasticity. The authors write that they average 50 simulations, which raises a few questions

a. Why not include standard error/standard deviation in their figures? Only showing the average does not represent the full range of possible outcomes and makes it difficult to evaluate the impact of stochasticity in this system.

Thanks for the reviewer’s helpful comment. We totally agree with the reviewer. So, we added standard error bars on the figures in the manuscript and also added a sentence in the figures captions (**Figures 3, 4, 6, 7, 10,** and **13**).

b. When they report oscillations in their average values (e.g. Fig 8) – does that imply that all of the individual simulations oscillate at the same frequency and amplitude?

Thanks for the reviewer’s useful comment. We added a new figure (**Figure 9**) and showed that not all of individuals oscillate at the same frequency and amplitude. In addition, a paragraph was added for explaining this Figure in the “**Results and Discussion**” section (**pages 10** and **11**).

“Total number of different cell types versus time for five different samples are shown in Figure 9. As it is seen, not all of individuals oscillate at the same frequency and amplitude. In addition, one of the samples does not show oscillating dynamics and for this sample, the number of infected cells goes to zero very fast due to a small number of this cells at initial state (namely only one infected cell) and the number of T cells decreases slowly to a zero value due to the natural death. We removed eleven samples of that kind of situations from the measurement. Therefore, total number of different cell types versus time which were calculated by performing an average over 39 remaining samples, are shown in Figure 10 and their standard errors are shown by error bars.”

4. In the introduction, the authors said that T cells are initial targets. But healthy cells which can be infected and T cells are two different groups in their model. Infected cells with T cells around can change either to healthy cells or T cells. It's unclear what is the relationship (and the difference) between healthy cells and T cells in the model.

Thanks for the reviewer’s helpful comment. In the “**reaction step**” (second part, **page 6**), we explained that an infected cell with at least one T cell as its neighbor, will die and will be replaced by a T cell with *P_T_* probability, or turn to a healthy cell with (1- *P_T_*) probability in the next time step. Therefore, the more probability for creation of T cells, the more probable the replacement of an infected cell with a T cell. Otherwise, an infected cell will be replaced with a healthy cell. This selection rule is equal to the second term of Equation 1 in the manuscript (**page 4**). So, this would be a relationship between healthy cells and T cells in the CA model.

5. There are many different types if T cells with different functions. Please specify the type of T cell considered in this model.

Thanks for the reviewer’s useful comment. We added an explanation in the “**Material and Methods**” section (at the end of **page 4** and beginning of **page 5**) for clarification to a user.

“As it was mentioned in some experimental studies, the most noticeable responses were seen in CD8 T cells (over 50% on activation and proliferation). Therefore, when we talk about T cells in our model, in fact we consider CD8 types of T cells.”

6. In the results section a lot of different scenarios are tested, with different parameters and movement settings, which is good. But some problems exist:

a. Influence of PH and PD are specifically tested with different values, but it is unclear why those two are chosen, not PI and PT.

b. PI and PT are also changing from fig. 3 to fig. 8. But the change is not specifically mentioned. What is the influence of these parameters?

Thanks for the reviewer’s useful comment. In response to both comments “a” and “b”, we added explanations of the influences of *P_I_* and *P_T_* in the “**Results and Discussion**” section (**page 12**).

“The effect of an increasing value of *P*_I_ is shown in Figures 3, 4, 6, and 7D. For *P*_I_ larger than 0.3, the number of infected cells in the steady state reached to non-zero values and increased. Comparing Figure 7D with Figures 4 and 6 demonstrates that the number of healthy cells in this case at the steady state is much smaller than the previous cases and the number of infected cells increased. Comparing Figure7A with Figure 8 shows that increasing the value of *P*_T_ has not a significant effect on the number of infected and healthy cells in the steady state. The results show two important factors which are able to decrease the number of infected cells significantly; the values of *P*_I_ and *P*_D_. As shown in Figures 7-A and 8, for the case of *P*_I_ =1 (maximum value of contamination ability of infected cells) but the very small value of *P*_D_ (long lifetime of T cells), the number of infected cells in the steady-state goes to a very small value. By decreasing the lifetime of T cells, the number of infected cells in the steady state increases (Figures 4 and 7). Increasing the value of the probability of death of infected cells (*P*_H_) has not a significantly effect on the number of infected cells in the steady state. It should be noted that in Figure 6-C the value of *P*_H_ is very larger than the value of *P*_I_ nevertheless the number of infected cells does not go to zero in the steady state.”

c. In fig. 6 C, when the death rate of infected cells is high, instead of infected cells, T cells die out. This is quite counter intuitive and warrants more discussion. Also, it is surprising that infected cells can be maintained at low level in the absence of T cells.

Thanks for the reviewer’s helpful comment. We added explanations in the “**Results and Discussion**” section (**page 9**).

“In all cases, the number of infected cells increased to reach into a steady state. The number of healthy cells shows a rapid growth at the beginning of the automata due to a rapid decrease of the number of T cells because of the large value of *P*_D_. After 25 time steps, each infected cell was surrounded approximately by four healthy cells which is the cause of a large number of the healthy cells. Although the value of *P*_T_ is greater than the value of *P*_I_, the number of T cells is low and a chance of a T cell to come close to infected cells is small. So, the number of T cells increases slowly. By increasing value of the probability of the natural death of the infected cells (*P*_H_), the chance of a T cell to join an infected cell before its death decreases. As shown in Figure 6-C, in the case of *P*_H_=0.9, the number of the T cells turned to a near zero value after initial decrement and then an increment very smoothly to reach into a steady state after about 300 time steps.”

d. There are lots of details given describing what the figures show, but this type of discussion distracts from the main idea that's being delivered. There should be some more conclusive statement summarizing/synthesizing the findings from each scenario.

Thanks for the reviewer’s useful comment. We agree with the reviewer and we tried to apply some changes by adding/rewriting some sentences includes better explanations and adding three new figures (**Figures 9, 10,** and **11**) in order to make the “**Results and Discussion**” section more conclusive for each scenario (for example, **pages 7, 8, 9, 10, 11, 12,** and **13**).

e. The oscillations are a key feature of their results, but an explanation of the source of oscillations is lacking.

Thanks for the reviewer’s helpful comment. We explained how the waves are generated (**page 11**) in the “**Results and Discussion**” section and also added a new figure (**Figure 11**).

“The snapshots of one of the samples which are taken at every 20 time steps are shown in Figure 11. The simulation started by inserting one infected cell in the centre of the lattice. In this case, we set *P_I_*=1 which means the contamination ability of the infected cells is at its maximum value. The ability of movements of the cells makes the chance to the infected cells to come close to the healthy cells and convert them to the infected cells. In contrast, the number of T cells will increase in the centre of the lattice because of the value of the probability of creation of a T cell is much more than the value of the probability of their death. These two mechanisms make the possibility of formation of moving circular waves with infected cells at their boundary and large numbers of T cells at the inner layers. By passing time, the size and the number of these waves decrease and increase respectively.”

7. The second paragraph in the results section mostly summarizes their results again. Some of this discussion would be better included in the results section as it doesn’t really help the reader connect their findings to the broader field.

Really thanks for the reviewer’s useful comment. We totally agree with the reviewer. Therefore, we rewrote most parts of the “**Results and Discussion**” section (for example, **pages 7, 8, 9, 10, 11, 12,** and **13**) and added three new figures (**Figures 9, 10,** and **11**) in order to help the reader understand the findings better.

Here are some other changes we made in the revised version of the manuscript:

- In the “**Material and Methods**” section (**page 5**), we corrected the value for the probability of randomly distributed cells (from 10% to 50%). In addition, based on the explanations we added to the manuscript, we replaced **Figure 2** with the newer one in the revised manuscript.
- While adding standard error bars in the Figures, we found an error in setting parameters through one of our codes that generated **Figure 3** (“**Results and Discussion**” section). Therefore, we corrected the parameters values and replaced **Figure 3** with the corrected one and corrected the explanations regarding that Figure in the manuscript text as well (**page 7**).
- We have replaced two words with ones with better meaning in “**Introduction**” section, **page 2**.

At the end, we thank the referees for reviewing our manuscript which helped us to significantly strengthen our manuscript. We hope that our responses to the comments and the improved version of the manuscript would be in accordance with the reviewers’ opinions.
