# Supplementary material for: On the possibility of oscillating in the Ebola virus dynamics and investigating the effect of the lifetime of T lymphocytes
Source: PLoS One. 2022 Mar 11;17(3):e0265065. doi: 10.1371/journal.pone.0265065 (PMC8916666; doi:10.1371/journal.pone.0265065)
Supplement: Attachment — Submitted filename: Response to Reviewers.docx [file pone.0265065.s003.docx]

Please include the following items when submitting your revised manuscript:

A rebuttal letter that responds to each point raised by the academic editor and reviewer(s). You should upload this letter as a separate file labeled 'Response to Reviewers'.

We have responded to the reviewers’ comments point-by-point. The reviewers' comments are in red. Our responses have been written as black text.

Reviewer #1: The authors have made significant updates to improve the manuscript. Two minor comments based on their revisions are below.

We are grateful for the time and effort spent by the reviewer on this manuscript, which helped us to significantly strengthen our manuscript through a detailed addressing of their concerns.

The addition of figure 9 is very helpful. The authors explain the cases where the infected cells go to zero as being because of starting with only one infected cell. But don’t all simulations start with one infected cell?

Thanks for the reviewer’s helpful comment. Although all the samples have the same number of healthy and T cells and one infected cell at the center in their initial state, for each sample a different random seed is used, and the initial location of T cells differs for different samples. So, due to this randomness, in some samples the number of infected cells might go to zero very fast.

We added explanations in the “**Material and Methods**” section (**page 5**).

It is still not clear to me what the parameter PT represents and what it means biologically. Does it represent T cell proliferation in response to interaction with an infected cell? Does (1-PT) represent regeneration of the dead target cell?

Thanks for the reviewer’s useful comment. *P*_T_ represents the probability of T cell proliferation in response to interaction with an infected cell and (1-*P*_T_) represents the probability of the replacement of a dead target cell with a healthy cell.

We added explanations in the “**Material and Methods**” section (**page 6**).

At the end, we thank the referees for reviewing our manuscript which helped us to significantly strengthen our manuscript. We hope that our responses to the comments and the improved version of the manuscript would be in accordance with the reviewers’ opinions.
